# Tango Decorations at Leeds

**Published:** 1914-01-03

**Authors:** 


### Tango Decorations at Leeds.

THE interior of the Women and Children's Hospital, Leeds, presented a gay and festive appearance on Christmas Day. The decorations formed a very pretty sight. The corridor lights had red shades, and in the entrance hall was a large Christmas-tree. Alexandra Ward was decorated to represent autumn, and it looked very pretty and light; the centre lights had shades of cream paper, finished at the bottom with gold-bead trimming and autumn leaves hanging from the ceiling and on the shades; the side lights over each bed had pretty old-rose lights, and ivy and autumn leaves were again applied on the brackets, festoons of ivy and autumn leaves were across and around the ward. The flowers were bronze and white chrysanthemums and large white lilies.

Victoria Ward was most up to date in its tango dress, and looked very pretty and effective when lighted up, with festoons of coloured leaves and evergreens and tiny Chinese lanterns. The flowers were bronze chrysanthemums, pink carnations, and roses, and some fine palms. The maternity wards were decked out in violet and silver, and a very charming and novel picture they looked; the cots were tied up with pale mauve ribbons, and the flowers were white and green spring flowers. The patients each received a gift of useful clothing and a toy, sent by the West Riding Needlework Guild, various clubs, and ladies interested in the hospital. The Christmas dinner was much enjoyed by the patients, the fare including turkey and plum-pudding, followed by dessert. During the afternoon each patient was allowed a visitor to tea from two to five o'clock. On Tuesday and Wednesday the patients were entertained by the nurses, both the day and night staff having arranged entertainments. The domestic staff had their dinner on Christmas Day and the nursing staff had theirs in the evening, followed by an impromptu dance. The nursing and domestic staffs will have their annual dances early in January.

